# Mechanistic insights into facet-dependent CO adsorption and vibrational responses on anatase TiO_2_: periodic quantum-mechanical calculations

**DOI:** 10.1007/s00894-026-06703-w

**Published:** 2026-04-13

**Authors:** Jaroslav Vacek, Pavel Hobza, Dana Nachtigallová

**Affiliations:** 1 Institute of Organic Chemistry and Biochemistry, Czech Academy of Sciences, Flemingovo námĕstí 542/2, 16000 Prague, Czech Republic; 2 IT4Innovations, VŠB−Technical University of Ostrava, 17. Listopadu 2172/15, 708 00 Ostrava-Poruba, Czech Republic; 3 Department of Physical Chemistry, Palacký University Olomouc, tř. 17. Listopadu 12, 771 46 Olomouc, Czech Republic

**Keywords:** DFT, Molecule-surface interactions, TiO_2_ anatase, CO IR probe, Surface topology, Density functional calculations, Surface chemistry

## Abstract

**Context:**

The study presents a comprehensive computational analysis of CO adsorption on anatase TiO_2_ surfaces with the (001) and (111) facets, utilizing CO as an IR probe. CO adsorption orientations and binding strengths vary between surfaces due to differences in local surface geometries and Ti coordination environments. Detailed electronic structure analyses, including density of states and wavefunction visualizations, show that σ-donation dominates on the (111) [TiO_3_] site. At the same time, π-backdonation is more prominent at the (111) [TiO_5_] site, correlating with a blue shift of the CO stretching frequency for the (111) [TiO_3_] site and a red shift for the (111) [TiO_5_] site. Surface oxygen atoms are key contributors to π-backdonation. The results highlight the critical role of surface topography and coordination environment in governing CO adsorption behavior, extending previous insights into anatase surface chemistry.

**Methods:**

All periodic calculations were performed using the Vienna ab initio package (VASP), employing the GGA-PBE functional and a plane-wave basis set, with the projector-augmented wave (PAW) method for the description of core electrons. All cluster calculations were performed using the ORCA package. We employed the PBE and B3LYP functionals, as well as the MP2 wavefunction–based method, all with the TZVP basis set. For the localized average ionization energy (ALIE), we employed the CCSD(T)/aug-cc-pV5Z level of theory.

**Supplementary Information:**

The online version contains supplementary material available at 10.1007/s00894-026-06703-w.

## Introduction

**Fig. 1:**
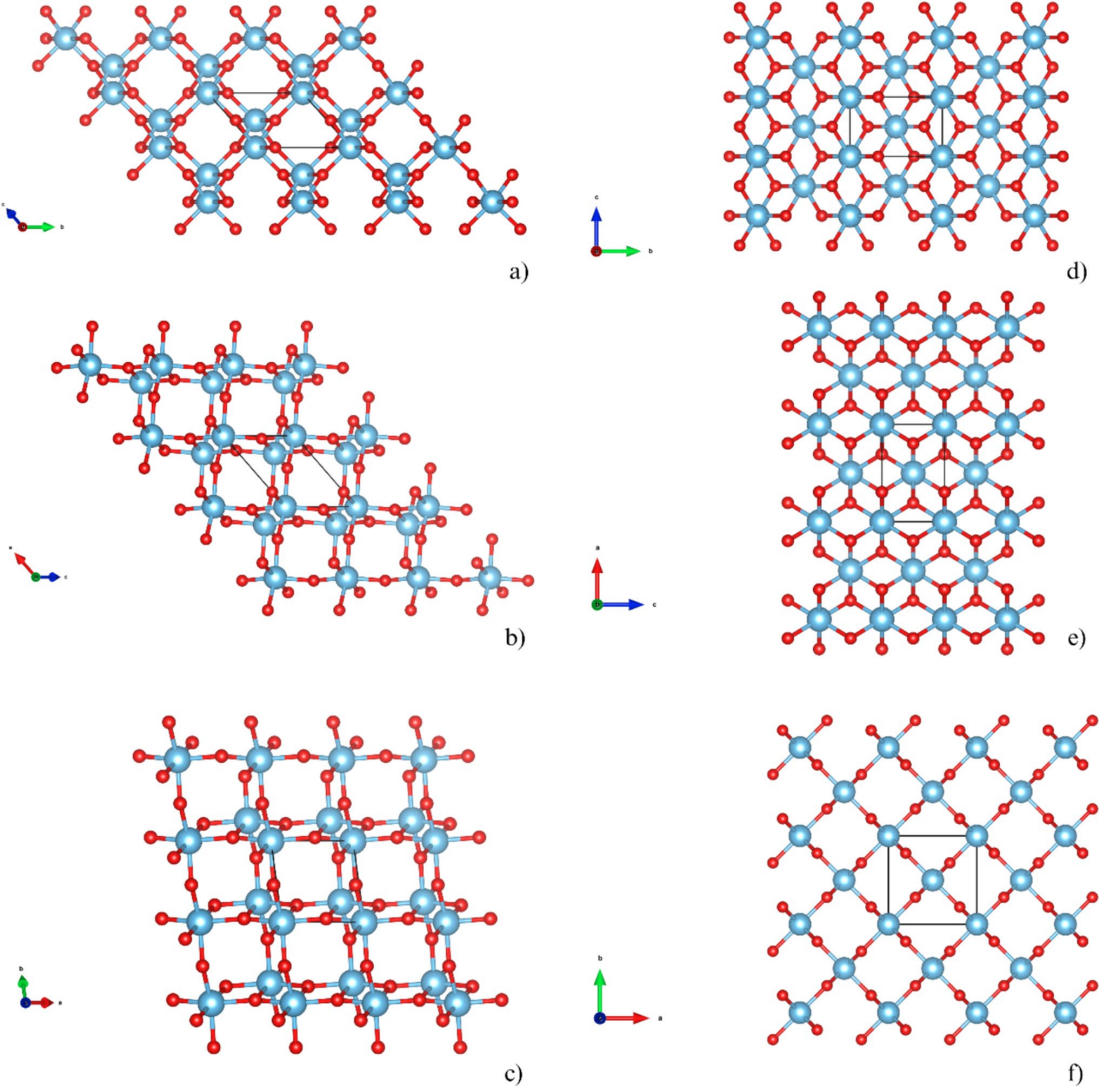
The bulk structure of the two photoactive phases of TiO_2_, anatase (**a**–**c**) and rutile (**d**–**f**), as viewed along three crystallographic directions (red oxygen atoms, blue titanium atoms). The data were obtained from X-ray scattering experiments [28, 29]. The primitive unit cells are indicated. In anatase, the primitive cell is triclinic (due to its minimal volume representation), while rutile’s primitive cell is tetragonal. However, the overall crystal symmetry of both phases is tetragonal

Since the discovery of the activity of TiO_2_ in the light-induced decomposition of water into H_2_ and O_2_ and CO_2_ reduction, research on TiO_2_ has grown considerably [1–15]. Its popularity stems from its chemical stability, non-toxicity, natural abundance, ease of synthesis, and low cost. However, despite these advantages, TiO_2_ still lacks specific critical properties, such as an optimal band gap and effective charge separation, which limits its performance [16]. To overcome these challenges, current research is focused on enhancing the efficiency of TiO_2_ through various modifications, including doping, functionalization, and defect engineering [17–19]. Among the TiO_2_ crystalline phases, anatase and rutile have been the most extensively studied (Fig. 1).

Anatase features a variety of crystallographic surfaces, each characterized by unique Miller indices. The (101) surface is the most thermodynamically stable and is primarily observed. However, advancements in crystal engineering have made it possible to produce anatase crystals with higher-energy surfaces. Reference [20] was the first to present the synthesis of TiO_2_ anatase nanocrystals with the (111) facet exposed and provided evidence of this via high-resolution TEM microscopy. Several experimental papers have been published providing additional information on the existence of the (111) surface [21–23]. The surface energy of these surfaces follows the order (111) > (001) > (101) [20]. Nanocrystals of TiO_2_ anatase with exposed (001) and (111) facets have been shown to be highly reactive [24]. Furthermore, the (111) facet shows higher photoactive selectivity than the (001) surface [25]. Despite significant progress, the exact mechanism underlying water splitting and the activity among different surfaces remains not fully understood [26, 27].

Existing research has shown that facet engineering, which encompasses various TiO_2_ facets, can enhance photocatalytic activity. In this regard, the photocatalytic activity of the anatase (111) surface remains underexplored compared to the widely studied (101) and (001) facets. However, to gain a comprehensive understanding of the photocatalytic mechanism and improve its efficiency, it is crucial to investigate the surface structure of the catalyst thoroughly. One effective approach involves using probe molecules, whose experimentally measurable properties can vary significantly, reflecting differences in interaction with surface sites. Among these, carbon monoxide (CO) is a commonly utilized probe molecule, well known for its sensitivity to the local chemical environment through shifts in its vibrational stretching frequency (ν_CO_), which are highly responsive to changes in the chemical context and occur in a spectral region well separated from the framework vibrations (the experimental value of the fundamental vibrational stretching frequency is 2143 cm^−1^ [30]), making them easily distinguishable and readily detected using infrared (IR) spectroscopy [31–34].

The sensitivity of ν_CO_ arises from the unique electronic structure of the CO molecule, which enables it to act as both an electron acceptor (Lewis acid) and donor (Lewis base). Upon adsorption onto a surface, CO interacts with the surface through (i) electrostatic interactions, (ii) the donation of electron density from the highest occupied molecular orbital (HOMO), specifically the antibonding σ-orbital, to the surface, and (iii) π-backdonation, involving the transfer of electron density from the metal center into the lowest unoccupied molecular orbital (LUMO), the antibonding π-orbital [17]. These interactions coexist, and the resulting shift in ν_CO_ frequency reflects a delicate balance between them. For TiO_2_ surfaces, studies have shown that electrostatic interactions and σ-donation (mechanisms (i) and (ii)) predominantly govern CO adsorption at fivefold-coordinated titanium [Ti(5)] sites on both (110) rutile and (101) anatase surfaces [27, 35–37]. This dominance is evidenced by increased ν_CO_ values, which correlate with the degree of Ti-site unsaturation. A lower correlation facilitates greater electron withdrawal, as this site becomes more reactive. The withdrawal of electron density from the antibonding HOMO of CO strengthens the bond, resulting in a blue shift of ν_CO_. Consequently, one might observe even larger ν_CO_ shifts at fourfold-coordinated sites [Ti(4)]. Indeed, multiple previous studies suggest that this assumption is valid [34, 37–44]. However, in reference [31] it is reported that CO adsorption on [TiO_4_] sites of anatase (110) surfaces exhibited lower ν_CO_ values compared to [TiO_5_] sites on anatase (101) surfaces. This counterintuitive observation was attributed to backdonation from adjacent surface oxygen atoms, which donate electron density into the LUMO (2π* orbital), weakening the CO bond and causing a red shift in ν_CO_. Therefore, the donation/backdonation should be regarded as a property of the surface as a whole, rather than the character of the individual local site.

Numerous computational studies on the surface of anatase’s behavior have been published, utilizing periodic calculation methods. These studies have examined the adsorption of water [45, 46], formaldehyde [47], formic acid [48], ethanol [49], and the CO molecule [31, 37, 50, 51] on both the (101) and (001) surfaces. Noticeably, reference [37] presents a multi-technique study on the adsorption of CO molecules on the anatase (101) surface, demonstrating good agreement between experimental IR spectroscopy and periodic DFT calculations, as well as reasonable agreement between temperature-programmed desorption data and DFT results. The close match between the theoretically predicted and experimentally observed CO stretching frequencies (ν_CO_) confirms the reliability of periodic DFT calculations. Reference [52] reports a correlation between the binding energy of a CO molecule and the blue shift of its stretching vibration frequency on anatase (101) and (001) surfaces. In this work, we present the results of cluster and periodic calculations aimed at characterizing the (111) surface of anatase, a facet that has not been extensively studied to date. For comparison, we also examine the well-characterized (001) surface of anatase. To this end, we model key properties, focusing particularly on the vibrational frequencies of a CO probe molecule adsorbed at various sites on the anatase surfaces. Our research on the high-energy (111) anatase surface reveals significant deviations from its ideal geometry after optimization, suggesting that the actual structure of high-energy anatase surfaces may differ from conventional idealized models. We also demonstrate how the structure, particularly the coordination of Ti atoms, is reflected in the stretching frequencies of CO molecules, which are often used as probes for analyzing the local surface structures.

## Methods

**Fig. 2:**
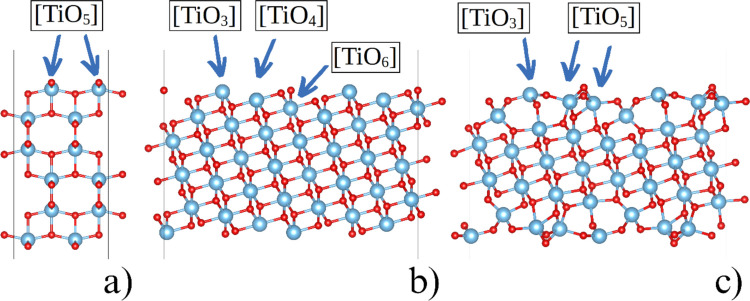
The periodical models of the (001) (**a**) and (111) (**b**) surfaces as cut by the respective lattice and the optimized (111) surface (**c**). Note the significant change in the geometry of the surface atoms of the slab in comparison to the ideal (111) surface (**b**) vs (**c**)

Two TiO_2_ anatase surfaces, (001) and (111) (Fig. 2), taken from an experimentally obtained bulk anatase structure [29], were optimized using the periodic slab models, which were separated by a 20 Å vacuum layer. All unit cells were orthorhombic. The surfaces were stoichiometric anatase slabs, each approximately 12 Å thick, with symmetrical surfaces on both ends (see SI).

All periodic crystal calculations were carried out using the Vienna ab initio simulation packet (VASP), employing a plane-wave basis set and the projector augmented wave method to account for the distribution of the core electrons [53–56]. Both GGA (PBE [57]) and hybrid DFT (B3LYP [58]) functionals, augmented with the Grimme empirical dispersion with the Becke-Johnson damping function [53–55, 57, 59–61], were used.

The reliability of the periodic calculation was validated by computing the CO properties adsorbed on the anatase surface using the CO-Ti-(O-Ti)_*n*_-O_X_ clusters (Fig. S1), which model the (111) anatase surface, where *n* is either 3 or 5, and X denotes the number of terminating oxygen atoms required to maintain the stoichiometry. The anatase models were derived from the optimized crystal calculations, with only the C-O distance being optimized. Calculations were performed using GGA (PBE) and hybrid functionals of DFT (B3LYP) as well as wavefunction-based MP2 method employing TZVP basis set. The cluster calculations were performed via the ORCA calculation packet [62, 63].

The electrostatic potential (EPS) was calculated for the periodic models using a dense K-point mesh and processed by the Multiwfn—a multifunctional wavefunction analyzer [64, 65]. The resulting data were visualized by the VMD viewer [66].

The average local ionization energy (ALIE) was computed for an isolated CO molecule employing the ORCA package on [67]. As has been demonstrated in ref. [68], the CO molecule represents a challenging system in terms of accurately describing the charge distribution; therefore, a high level of theory is required. Accordingly, the calculations were performed at the CCSD(T)/aug-cc-pV5Z level of theory [68, 69]. The molecular geometry was fully optimized, and the resulting wavefunction was analyzed using the Multiwfn tool. The ALIE isosurface was subsequently visualized with the VMD program.

Analysis of the CO molecule vibration for all models was conducted using the partial Hessian vibrational analysis (PVHA) [70]. We only considered the atoms of the CO molecule and a small part of the framework, specifically the binding Ti atom and all framework oxygens bound to it. We performed frequency calculations based on the harmonic oscillator approximation level of theory. Additionally, we manually visualized all vibration modes to ensure there was no unwanted coupling between the CO vibration and the framework vibrations.

The valence electronic structure of the CO-surface complex was explored using the density of states (DOS) and projected density of states (PDOS) [71]. The DOS data were analyzed using the VASPKIT program [72].

## Results and discussion

### Periodic calculations on the crystals of (001) and (111) anatase

The optimization of the slabs with the (001) (Fig. 2a) surface resulted in minimal structural changes compared to the ideal configuration, with no alterations in the coordination of Ti atoms and a total relaxation energy of only −0.49 kcal mol^−1^ per TiO_2_ unit (i.e., the system is stabilized). In contrast, the geometry of the slab with the (111) surface underwent significant changes. Initially, the pre-optimized slab contained three, four, and six-coordinated atoms in the 1:1:1 ratio (Fig. 2b). During relaxation, the four- and six-coordinated surface Ti atoms shifted toward each other, leading to a new arrangement consisting solely of three and five-coordinated surface atoms, in a 1:2 ratio (Fig. 2c). The relaxation energy was −18.52 kcal mol^−1^ per TiO_2_ unit, indicating substantial structural change.

When studying surface models with high surface energy, it is essential to account for the effects of cell size and periodicity [73]. Reference [74] focuses on the water-induced reconstruction on the anatase (001) surface and demonstrates how the size and shape of the supercell influence the formation of defects. In our case, the chosen supercell proves adequate; we tested several unit cells of varying shapes and sizes. All slabs and the vacuum layers had the same thickness. All models were fully optimized. As shown in Table S1, a 2 × 2 orthorhombic cell is sufficient, since the reconstruction energy per TiO_2_ unit is comparable to that obtained from larger supercells.

To obtain deeper insight into the electronic structure and surface reactivity, the electrostatic potential (ESP) was mapped on both surfaces using an isovalue of 0.001 e·bohr⁻^3^. The local minima and maxima of the ESP on each surface were identified using the Multiwfn program.

**Fig. 3:**
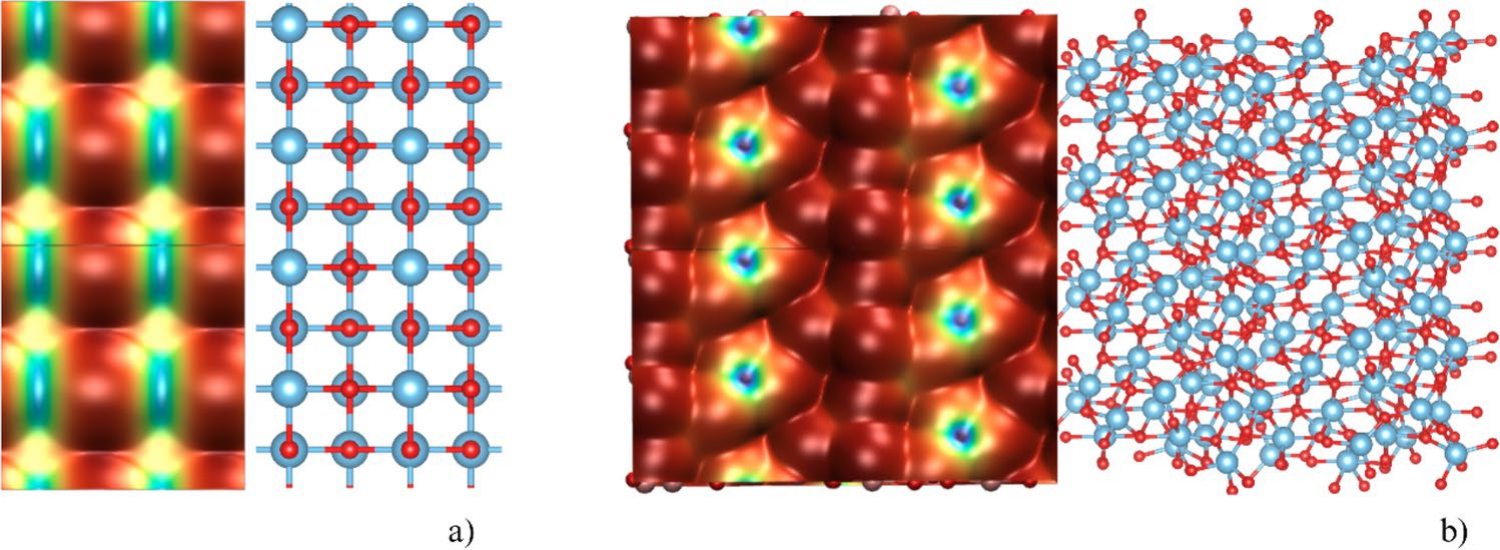
Top-down view of the (001) surface and its electrostatic potential (ESP) isosurface (**a**) and of the (111) surface and its electrostatic potential isosurface (**b**). The isovalue was set at 0.001 e·bohr⁻^3^. The color scale for the (001) surface ranges from 0.00 kcal·mol⁻^1^ (blue) to 4.28 kcal·mol⁻^1^ (red). For the (111) surface, the color scale spans from −10.52 kcal·mol⁻^1^ (blue) to 4.34 kcal·mol⁻^1^ (red)

On the (001) surface (Fig. 3), the positively charged five-coordinated Ti atoms appear as a grid of blue lines on the isosurface and are located in close proximity to negatively charged regions associated with surface oxygen atoms, promoting efficient π-backdonation. In contrast, on the (111) surface, positive ESP is localized at the three-coordinated Ti atoms, surrounded by a circular region of negative ESP, which hinders π-backdonation. Additionally, five-coordinated Ti atoms are not distinguishable, suggesting a strong π-backdonation capability of the sight. The absence of a distinct feature at the five-coordinated Ti sites of the (111) surface suggests that these positions are unfavorable for binding.

A comparison of the ESP maps of the two surfaces reveals a shift in the color scales: Specifically, the three-coordinated Ti atoms on the (111) surface exhibit substantially lower ESP (−10.52 kcal·mol⁻^1^) than for the (001) surface (0.00 kcal·mol⁻^1^). This difference suggests that the three-coordinated surface Ti atoms of the (111) surface are likely to serve as stronger binding sites than the five-coordinated Ti atoms of the (001) surface.

To access the reactivity of the CO molecule toward anatase surfaces, the localized average ionization energy (ALIE) was calculated, as it has been shown to correlate closely with chemical reactivity [67].

**Fig. 4:**
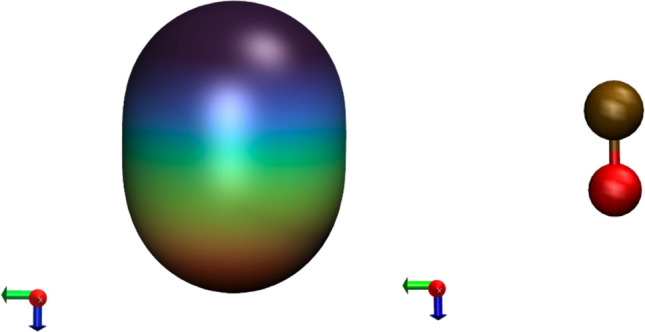
The average localized ionization energy (ALIE) of the CO molecule, shown together with the CO molecule itself to indicate orientation (red: oxygen, brown: carbon). The isovalue of the surfaces was set at 0.001 e/bohr^3^. The color scale ranges from 0.57 eV (blue) to 0.78 eV (red) to achieve an even distribution across the color scale

The ALIE of the CO molecule (Fig. 4) shows that the ionization energy reaches its minimum at the carbon atom and its maximum at the oxygen atom, indicating that the carbon atom serves as a nucleophilic center. This interpretation is supported by the atomic charge analysis, which assigns a negative charge to carbon and a positive charge to oxygen. As already noted, an accurate description of this distribution requires high-level theoretical methods, since lower levels of theory may yield qualitatively incorrect results [68]. Combined with the ESP surface maps, these results indicate that the CO molecule preferentially binds to surface Ti atoms via the nucleophilic carbon atom.

### Benchmark calculations

The performance of PBE and B3LYP functionals (see Table ST in the SI), used for periodic and cluster calculations, respectively, is tested by comparison with MP2 calculations. All methods predict a shortening of the bond relative to the bond length of the isolated CO molecule, with PBE slightly underestimating and B3LYP slightly overestimating the bond length. Furthermore, all approaches agree on a larger effect of binding in the case of three-coordinated Ti. A similar trend was also observed in the calculated CO vibrational frequencies, with all methods indicating a blue shift upon binding.

### Periodic calculations

The calculations on the periodic model are summarized in Table 1, which presents data on the orientation of CO adsorbed on TiO_2_ surfaces, characterized by the angles and close distances between the carbon and surface oxygen atoms. The results show that the regular structure of the (001) surface favors an almost perpendicular binding of CO, while a pronounced tilt is observed on the (111) surface. The number of surface oxygen atoms within 3.6 Å of the adsorbed CO is five, four, and three for the (111) [TiO_5_], (001) [TiO_5_], and (111) [TiO_3_] sites, respectively, with binding energies of 7, 15, and 24 kcal mol⁻^1^, increasing accordingly. These findings indicate that closely situated surface oxygen atoms destabilize CO. This trend is further supported by variations in Ti–C bond distances and shifts in the CO stretching frequency (ν_CO_). Notably, the CO bond elongates by 0.0033 Å and a red shift of 35 cm⁻^1^ occurs for the least stable (111) [TiO_5_] site, while bond shortening of 0.0010 and 0.0056 Å occurs, accompanied by blue shifts of 8 and 43 cm⁻^1^ for the (001) [TiO_5_] and (111) [TiO_3_] sites, respectively. The key characteristics of the CO–surface system (r_CO_ and ν_CO_) were verified using the more reliable B3LYP functional. At this level of theory, the isolated CO molecule has a bond length of 1.1311 Å and a stretching frequency of 2305 cm⁻^1^. The results follow the same trends as those obtained with the PBE functional: Adsorption of CO on the (001) [TiO_5_] site leads to a slight shortening of the CO bond (by −0.0029 Å) and a small blue shift (2 cm⁻^1^). When CO is adsorbed on the (111) [TiO_3_] site, the bond shortens more significantly (−0.0074 Å) and results in a large blue shift (25 cm⁻^1^). In contrast, adsorption on the (111) [TiO_5_] site leads to a notable elongation of the CO bond (0.0147 Å) and a substantial red shift (−64 cm⁻^1^). Thus, the two functionals are qualitatively consistent.

**Table 1:** The geometry parameters (Å), ν_CO_ vibrational frequencies (in cm^−1^), and binding energies (in kcal mol^−1^) of CO-TiO_2_ at the (001) [TiO_5_], (111) [TiO_3_], and (111) [TiO_5_] sites obtained from the periodic slab models and large cluster models. The B3LYP/SVP level theory was used for the cluster calculation

PBE								B3PLYP	
	r_CO_/Δr_CO_	ν_CO_/Δν_CO_	r_Ti–C_	r_O–C_	*E*_bind_	Θ_1_^[a]^	Θ_2_^[b]^	r_CO_/Δr_CO_	ν_CO_/Δν_CO_
(001) [TiO_5_]	1.1424/−0.0010	2134/8	2.302	2.676 2.676 3.335 2.380	15	91	92°	1.1278/−0.0029	2307/2
(111) [TiO_3_]	1.1378/−0.0056	2169/43	2.238	2.808 2.873 3.553	24	78°	58°	1.1233/−0.0074	2331/25
(111) [TiO_5_]	1.1467/0.0033	2091/−35	2.355	2.408 2.831 2.933 2.943 3.533	7	56°	76°	1.1457/0.0147	2243/−62

^[a]^ ∠(Ti-Ti-C)_x_, ^[b]^ ∠(Ti-Ti-C)_y_

The electronic structure of the systems is analyzed through changes in the electron density of states upon CO adsorption on anatase surfaces, complemented by the projected DOS method [71]. Furthermore, we present a visualization of the real parts of the electron band structure obtained from the periodic slab models. These band structures resemble the antibonding 5σ (HOMO) and antibonding 2π* (LUMO) orbitals of the isolated CO molecule, providing insight into the interplay between the σ- and π-back donations.

**Fig. 5:**
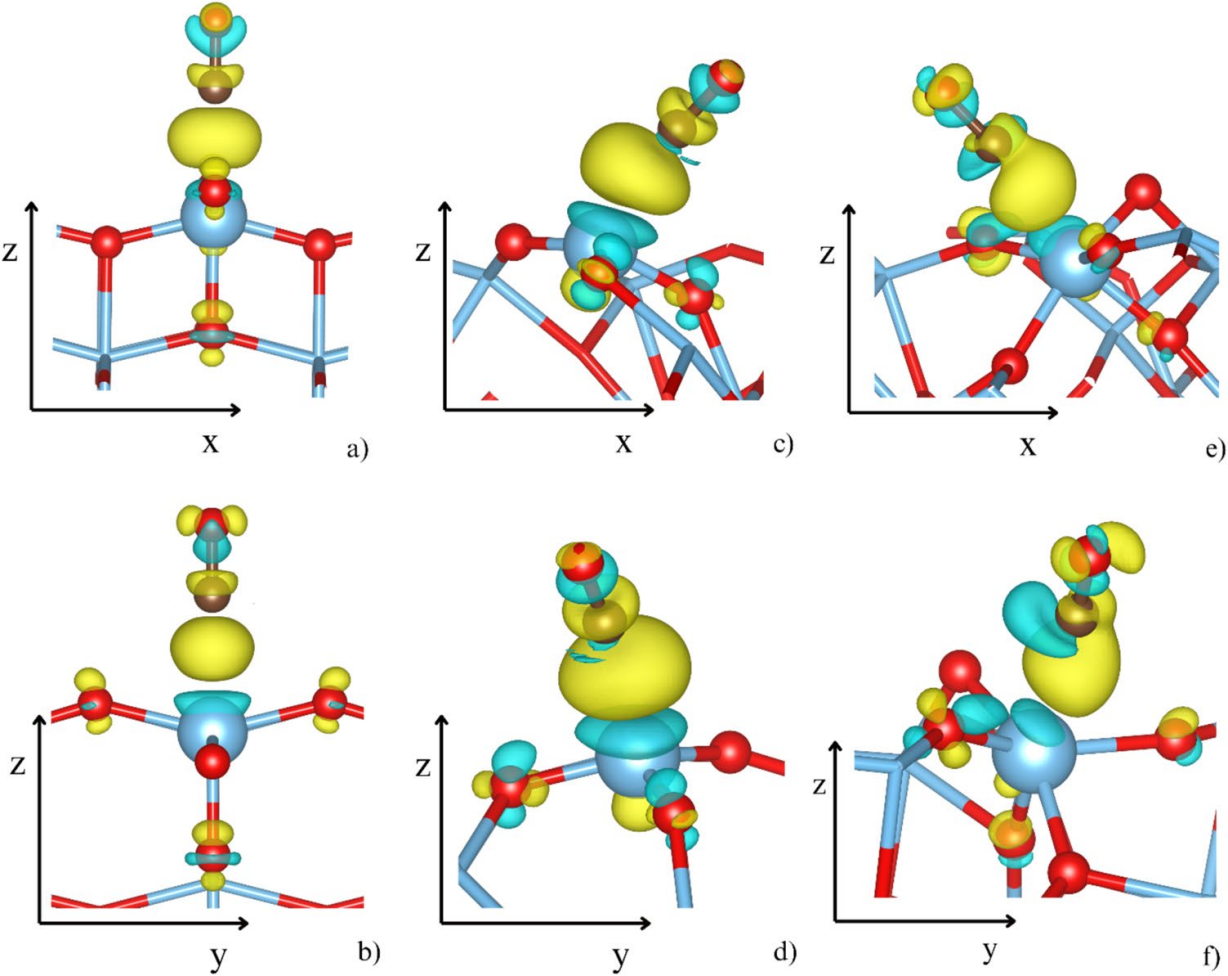
The difference between the electron density of the CO-surface complex and the isolated CO molecule and isolated surface for the CO-(001) [TiO_5_] (**a**, **b**), the CO-(111) [TiO_3_] (**c**, **d**), and the CO-(111) [TiO_5_] (**e**, **f**). Yellow and turquoise collars represent the regions with increased and depleted electron density, respectively (see Eq. S1 in SI)

Figure 5 presents the changes in electron density upon CO adsorption. The formation of the Ti–C bond leads to a localized accumulation of electron density, along with charges surrounding the CO oxygen and TiO_2_ oxygen atoms. Due to the inherent symmetry of the (001) surface, the CO-(001) [TiO_5_] site displays the most regular pattern of electron density redistribution. Specifically, there is a pronounced electron density accumulation at the Ti–C bond, a slight increase in density on CO oxygen atoms in orbitals perpendicular to the CO bond, and a depletion of electron density in orbitals aligned with the bond axis. Additionally, an enhancement in electron density occurs in some, though not all, surface oxygen atoms. At the CO-(111) [TiO_3_] site, a significantly larger accumulation of electron density at the Ti–C bond is observed, aligning with the strongest CO binding for this site. In this case, the electronic changes within the CO molecule are primarily confined to the σ-orbitals of the CO bond. In contrast, the CO-(111) [TiO_5_] site exhibits a more complex redistribution of electron density. While the overall increase along the Ti–C bond is like that of the CO-(111) [TiO_3_] site, it is accompanied by an asymmetric electron density depletion near a carbon atom. Furthermore, the changes in electron density of anatase oxygen atoms are also non-symmetric.

**Fig. 6:**
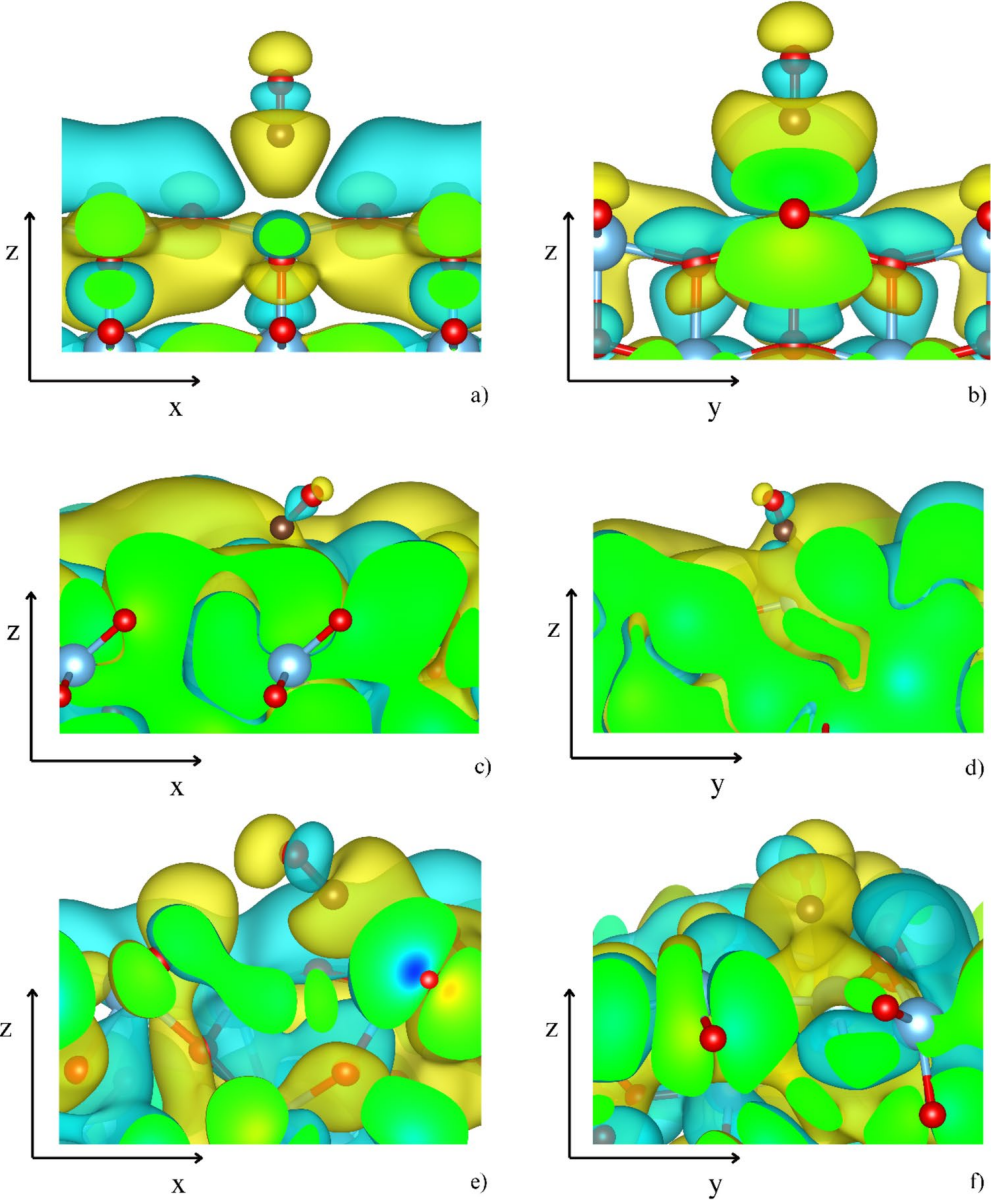
The real parts of the two-electron band structures reminiscent of the antibonding 5 σ HOMO orbitals from the periodic slab models of the CO-(001) [TiO_5_] (**a**, **b**), the CO-(111) [TiO_3_] (**c**, **d**), and the CO-(111) [TiO_5_] (**e**, **f**). The positive lobes are yellow, and the negative lobes are turquoise

**Fig. 7:**
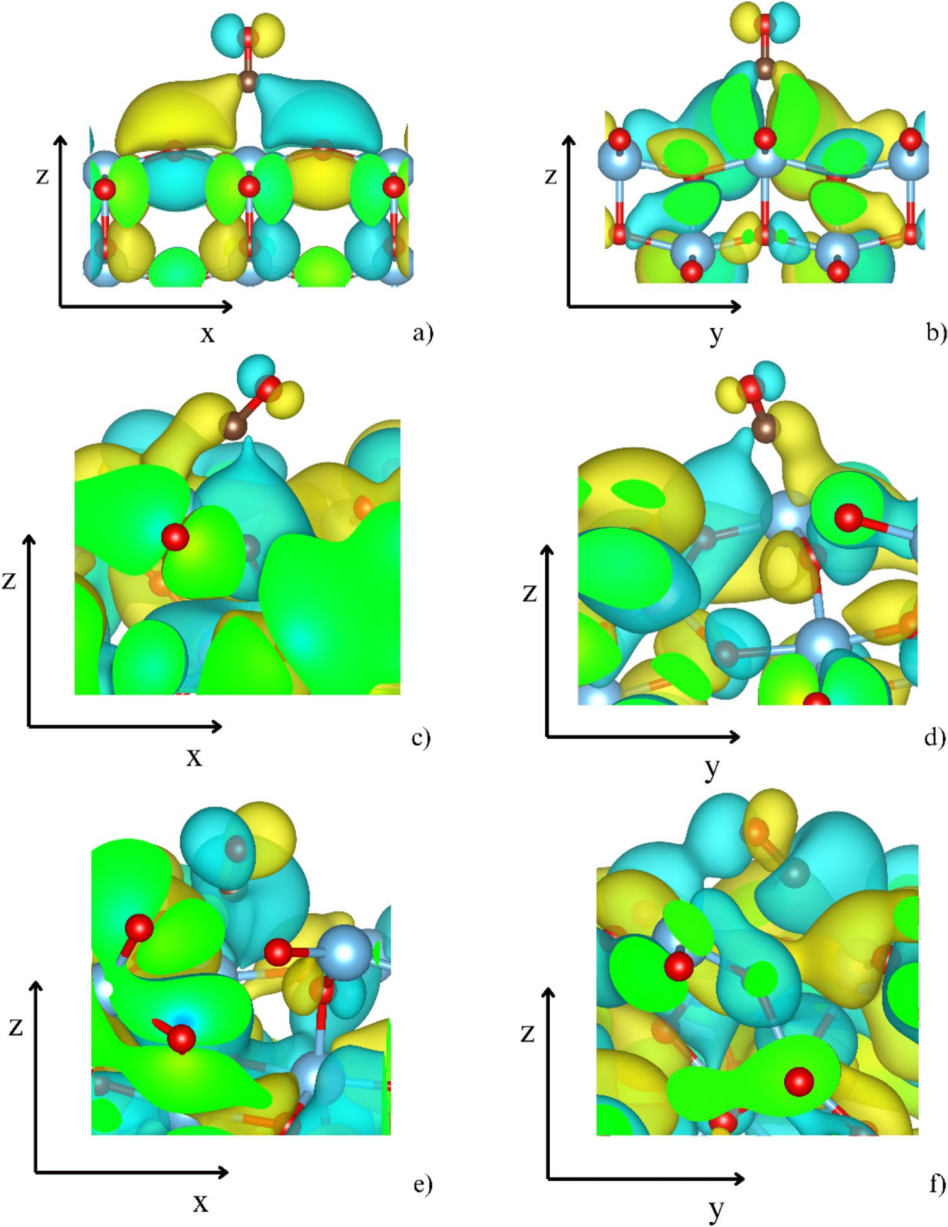
The real part of wave functions resembles π^*^(CO) antibonding orbitals at the (001) [TiO_5_] (**a** and **b**), (111) [TiO_3_] (**c** and **d**), and (111) [TiO_5_] (**e** and **f**) sites. The positive lobes are yellow, and the negative lobes are turquoise

The distinct electronic behavior resulting from the surface regularity of anatase and the orientation of CO relative to the surface is further evidenced by the visualization of the real part of the wavefunctions intersecting with the 5σ (HOMO, Fig. 6) and 2π_x_*/2π_y_* (LUMO, Fig. 7) orbitals. In the case of the (001) surface, a perpendicular orientation is reflected by a clear definition of the 5σ orbital, while it becomes highly delocalized on the (111) surface. This is particularly evident for the (111) [TiO_3_] site (Fig. 6c, d), where the 5σ orbital is barely distinguishable. Figure 7 displays the real part of the wavefunction associated with 2π_x_* and 2π_y_* of CO, showing well-defined orbitals at the (001) surface (Fig. 7a, b). Although the tilt angles of CO to the (111) surface are similar for the [TiO_3_] and [TiO_5_] sites—approximately 55° and 77°—the nature of the Ti-binding site differs markedly due to the different characteristics of the (111) surface. While the Ti atom at the [TiO_3_] site lifts slightly above the surface, it is embedded below the surface plane at the [TiO_5_] site. As a result, the 2π_x_* and 2π_y_* orbitals (Fig. 7c, d) are well defined at the (111) [TiO_3_] site with electron density contributions, similar to those observed at the (001) [TiO_5_] site. In contrast, at the (111) [TiO_5_] site (Fig. 7e, f), the wavefunction is more complex and exhibits significantly higher contributions, with a clear preference for the 2πy* orbital.

**Fig. 8:**
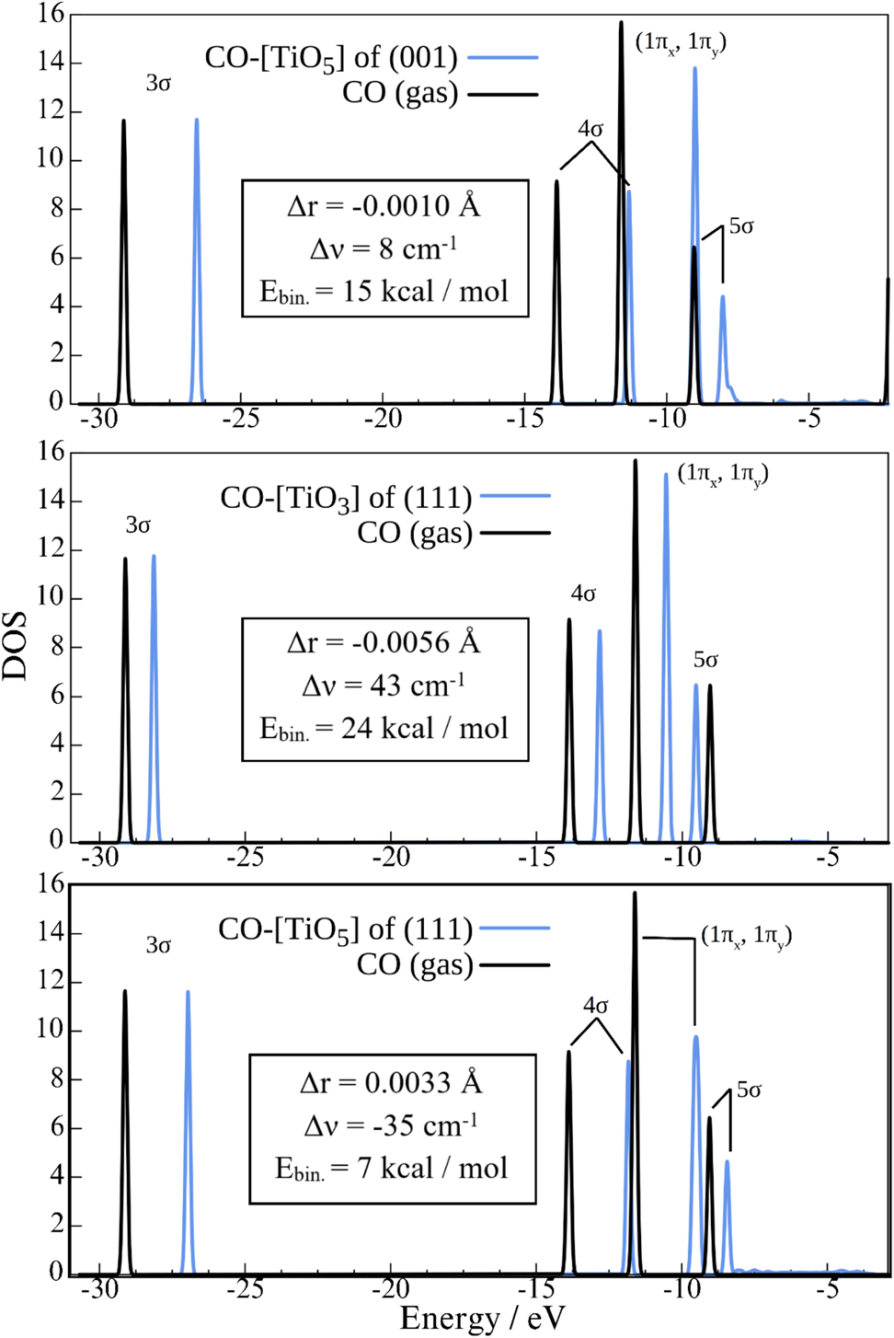
The density of states projected on the valence shell of an isolated molecule (black) and an adsorbed CO molecule (blue) on (001) [TiO_5_] site (top panel), (111) [TiO_3_] (middle panel), and (111) [TiO_5_] (bottom panel)

**Fig. 9:**
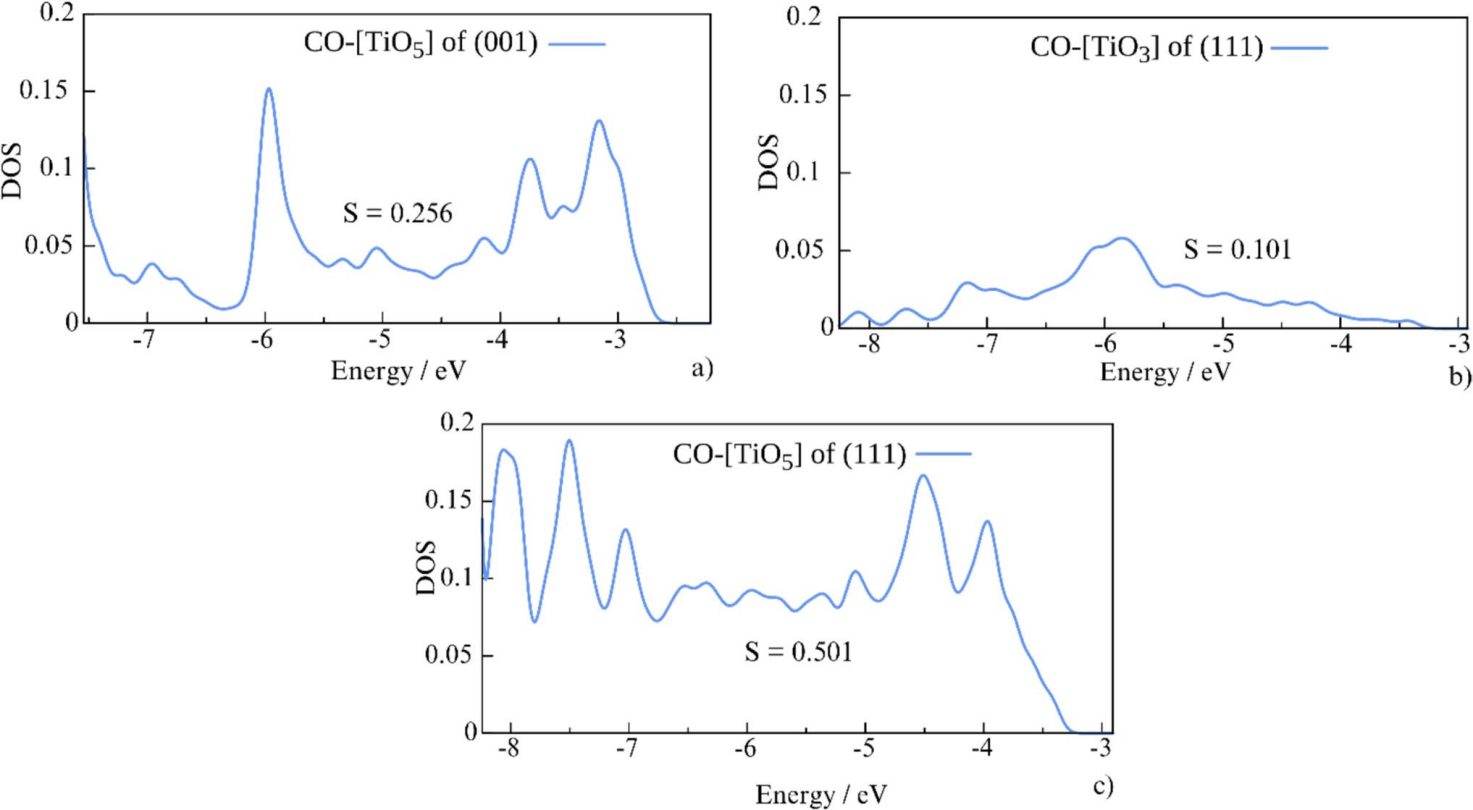
The projected density of states of the antibonding regions (from 5σ to E_Fermi_) of the adsorbed CO molecules on the (001) [TiO_5_] (**a**), (111) [TiO_3_] (**b**), and (111) [TiO_5_] (**c**) sites and the calculated surface area

Figure 8 shows the plot of DOS of the occupied valence orbitals, specifically bonding 3σ, antibonding 4σ and 5σ, and degenerate bonding 1π_x_,_y_ for all three systems compared to those of isolated CO. The plot extends to the Fermi level, at −2.21 eV (CO-(001)) [TiO_5_], −2.92 eV (CO-(111) [TiO_3_]), and −2.91 eV (CO-(111) [TiO_5_]). In every case, 3σ, 4σ, and 1π_x_,_y_ orbitals destabilize upon binding to the surface. This destabilization is more pronounced in the case of five-coordinated Ti sites when compared to CO-(111) [TiO_3_]. Importantly, the stabilization of the 5σ orbital is observed only in this scenario. Figure 9 provides more details in the vacant region of the systems, spanning from the 5σ energy level to the Fermi levels—an energy range that remains unoccupied in isolated CO molecules. The integrated areas of total DOS are 0.101, 0.256, and 0.501 for CO-(111)[TiO_3_], CO-(001)[TiO_5_], and CO-(111)[TiO_5_], respectively, aligning with the binding energies and spectral shifts of CO vibrational frequencies.

**Fig. 10:**
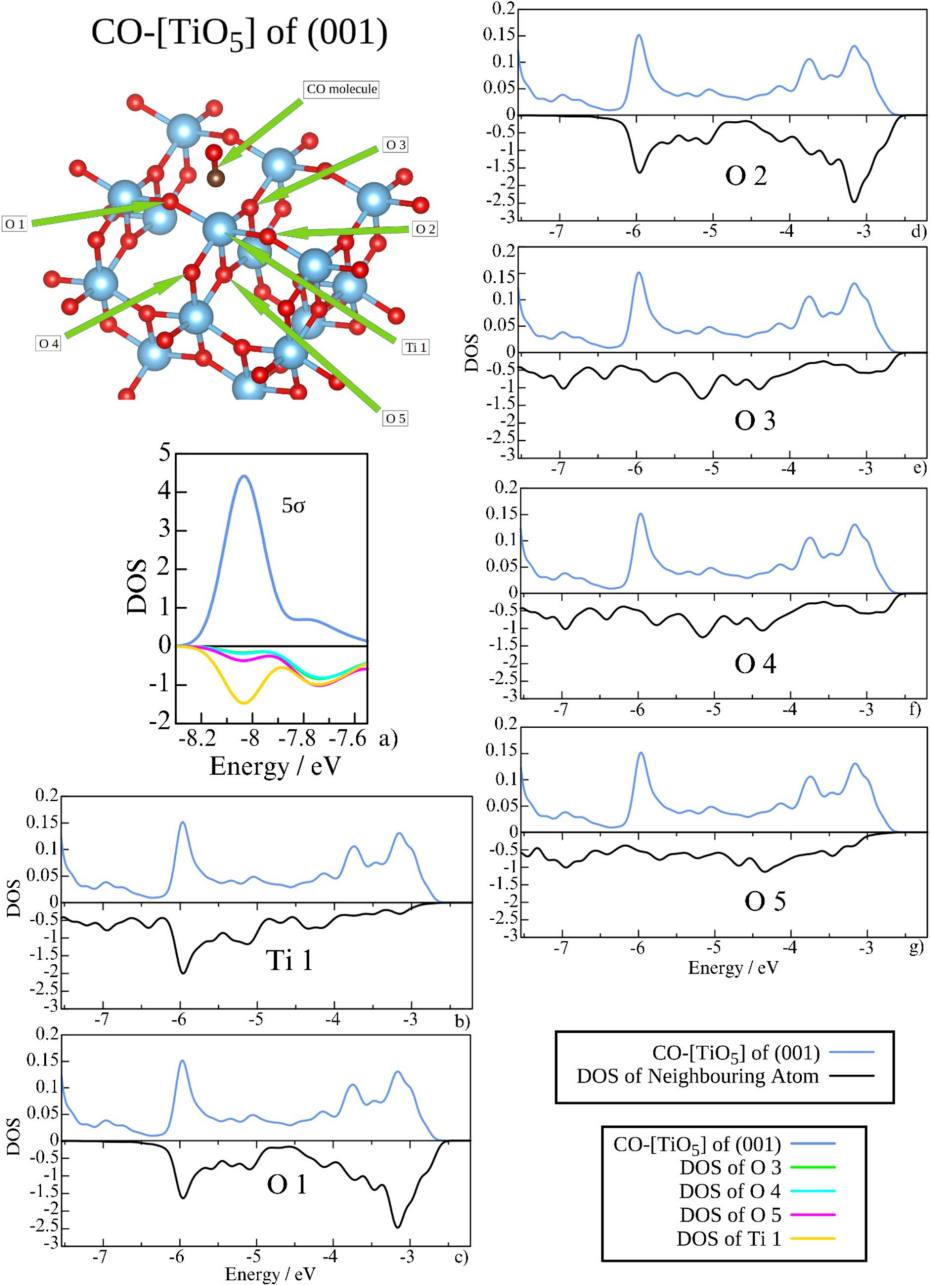
The projected DOS at the energy 5σ orbital (**a**) and antibonding region (**b**–**g**) of the CO molecule (blue) at the [TiO_5_] (001) site compared to Ti and adjacent surface oxygen atoms. Note that the scales of the plots are not the same for the CO molecule (positive) and the neighboring atoms (negative)

**Fig. 11:**
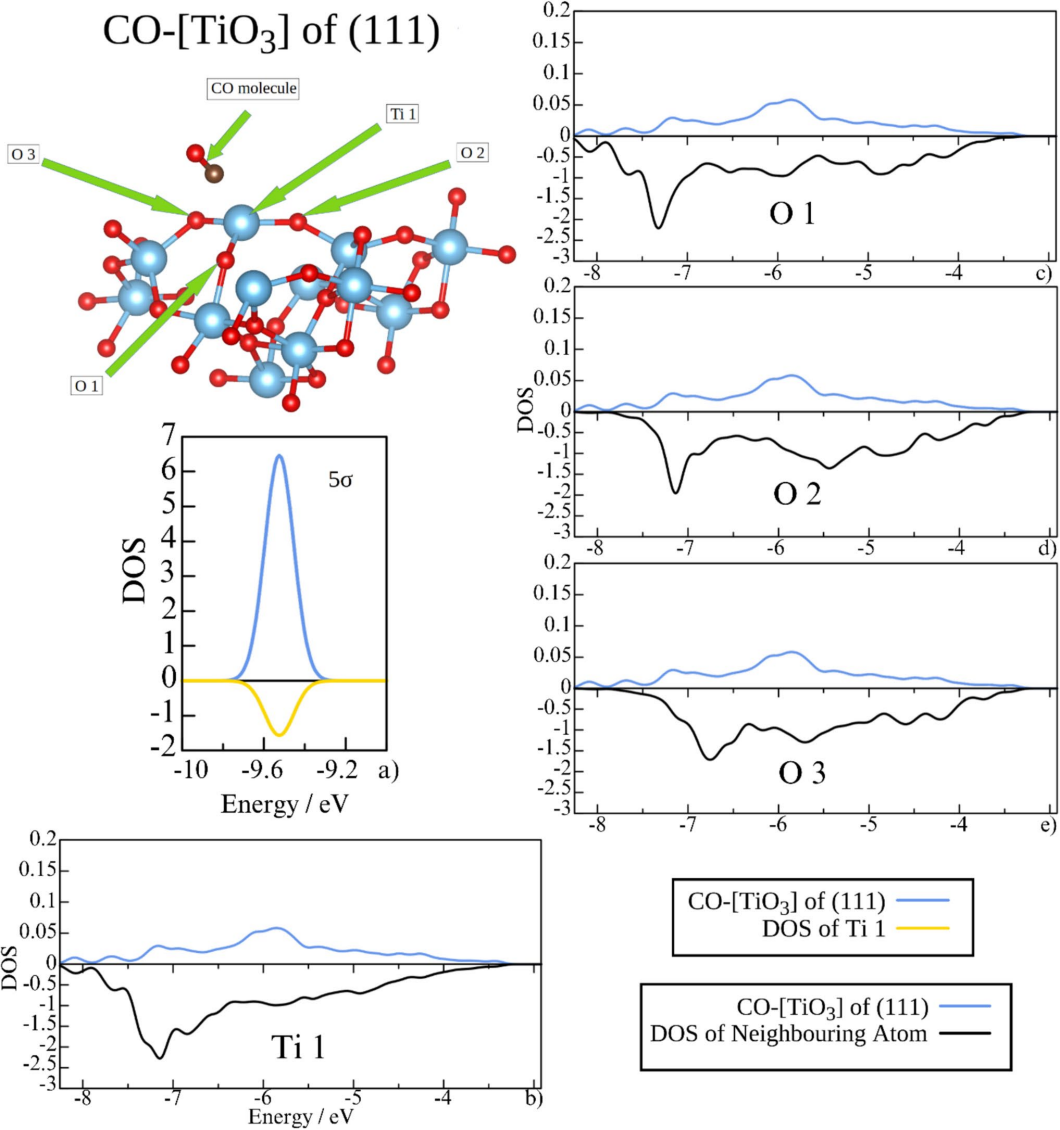
The projected DOS at the energy 5σ orbital (**a**) and antibonding region (**b**–**e**) of the CO molecule (blue) at the (111) [TiO_3_] site compared to Ti and adjacent surface oxygen atoms. Note that the scales of the plots are not the same for the CO molecule (positive) and the neighboring atoms (negative)

**Fig. 12:**
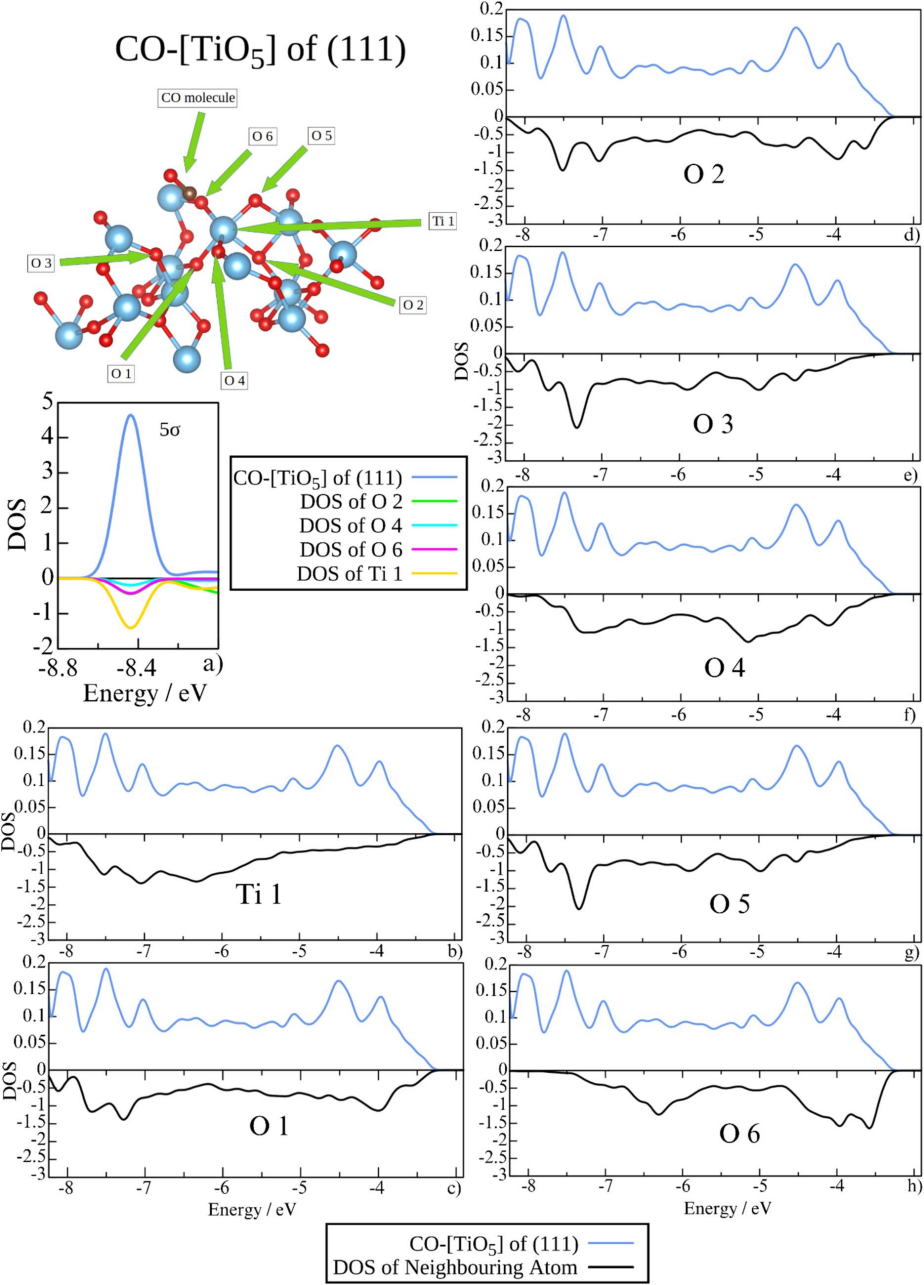
The projected DOS at the energy 5σ orbital (**a**) and antibonding region (**b**–**h**) of the CO molecule (blue) at the (111) [TiO_5_] site compared to Ti and adjacent surface oxygen atoms. Note that the scales of the plots are not the same for the CO molecule (positive) and the neighboring atoms (negative)

Figures 10, 11, and 12 offer a more detailed analysis of the backdonation mechanism in CO, emphasizing the contributions of individual surface atoms to the CO DOS. Generally, the occupancy of surface atoms in this energetic region is an order of magnitude greater than that of the CO molecule. For clarity and ease of comparison, we use different scaling for the DOS of CO and surface atoms. In all instances, the Ti-binding site contributes to the 5σ orbital, increasing its electron density and counteracting the stabilization of CO 5σ typically associated with σ-donation. This contribution is consistent across all sites. Interestingly, surface oxygen atoms contribute only at five-coordinated sites. This difference helps explain why the 5σ orbital is stabilized only in the case of the three-coordinated site. The electron density contributions at higher energies are attributed to the π-back donation.

In the case of CO adsorption on the (001) [TiO_5_] site, five surface oxygen atoms interact with the antibonding region of the adsorbed CO molecule. Due to symmetry, the density of states for O1 and O2, as well as for O3 and O4, is nearly identical. The contributions to the DOS of the 5σ orbital come from O3, O4, and O5 in addition to Ti. In contrast, O1 and O2 closely mirror the antibonding region of CO, indicating their key role in π-backdonation. This effect is illustrated by the electron density difference associated with CO adsorption (Fig. 5a, b). Furthermore, the interaction between CO and O1/O2 is reflected in the electron band structure, which resembles the antibonding 2π_x_* orbitals (Fig. 7a).

In addition to the absence of DOS at the energy of the 5σ orbital originating from surface oxygen atoms, the overall population of the antibonding region of CO on the (111) [TiO_3_] site is significantly lower (Fig. 9), even though all three adjacent surface oxygens overlap with the antibonding region of the adsorbed CO molecule (Fig. 11). Moreover, the DOS of those oxygens and that of Ti do not mirror the DOS of the CO molecule, indicating weak electron density sharing. The generally weak π-backdonation is further illustrated by the shape of the band structures corresponding to the LUMO orbitals of CO (Fig. 7c, d), which are asymmetric and poorly defined.

In contrast, the orientation of CO at the (111) [TiO_5_] site allows six surface oxygen atoms—one of which is not directly bonded to the Ti site atom—to contribute their DOS either at the energy of the 5σ orbital (notably O2, O4, and especially O6) or within the antibonding region of CO (Fig. 12). In this region, the overlap of CO aligns closely with the densities of O1, O2, and O3 states. Notably, at this site, the overall population of the antibonding region is the highest (Fig. 9).

### Comparison between different sites

The relative stability of the three studied systems follows this order: (111) [TiO_3_] > (001) [TiO_5_] > (111) [TiO_5_], which correlates with a progressive weakening of the Ti–C bond. At the (111) [TiO_3_] and (001) [TiO_5_] sites, the observed shortening of the C–O bond lengths and the corresponding blue shifts of the ν_CO_ stretching frequency are consistent with enhanced electron donation from the CO 5σ antibonding orbital. In contrast, although the ν_CO_ mode is blue-shifted in calculations for the small (111) [TiO_5_] cluster, the periodic calculations exhibit a red shift, indicating the significance of other effects originating from a larger surface surrounding. A more detailed analysis of DOS helps rationalize the observed differences. The most apparent spectroscopic and electronic signatures of increased σ-donation of CO bound to (111) [TiO_3_] are reflected in the stabilization of the 5σ(CO) orbital (Fig. 8), whose DOS overlaps solely with the Ti site atom (Fig. 11). In the antibonding region of the CO molecule, which is the smallest (0.101 states·eV⁻^1^·unit cell⁻^1^), there is minimal mirroring of the DOS between CO and those of Ti and surface oxygen atoms, underscoring the dominant role of σ-donation over π-back donation in this system.

DOS calculations further clarify the origin of the distinct spectroscopic signatures observed for CO adsorption on the [TiO_5_] of the (001) and (111) surfaces—specifically, the blue and red shifts in the ν(CO) stretching frequency, respectively. In both cases, the 5σ(CO) orbital is destabilized upon adsorption; this destabilization is particularly more pronounced on the (001) surface (Fig. 8), despite similar overlaps between the 5σ(CO) orbital and the Ti and surface oxygen states in both systems (Figs. 10 and 12). The main difference arises from the degree of electron population in the CO antibonding region, which is significantly greater for CO adsorbed on the (111) surface (0.501 vs. 0.256 states·eV⁻^1^·unit cell⁻^1^ for the (111) and (001) surfaces, respectively). This higher population correlates with a weakening of the C–O bond and the corresponding red shift in ν(CO), indicating more efficient π-backdonation. The difference in π-backdonation efficiency is further affected by the local geometry of the binding sites. On the (001) [TiO_5_] surface, CO binds nearly perpendicular to the surface, allowing for a well-defined symmetry and spatial alignment that restricts π-backdonation primarily to Ti and a few adjacent surface oxygen atoms. In contrast, on the (111) surface, CO adopts a tilted orientation, and the Ti center is more recessed into the surface topology. This configuration enhances π-backdonation from a broader network of surface oxygen atoms, contributing to the larger antibonding population observed.

These findings align with the previous study on CO adsorption at the anatase (101) [TiO_5_] and (110) [TiO₄] surfaces reported in reference [31], which emphasizes the critical role of surface topography, beyond just the coordination number of the Ti site, in determining adsorption properties. In particular, the results underscore the significant contribution of surface oxygen atoms as the dominant sources of π-backdonation in such systems.

## Conclusion

This study presents a detailed computational investigation of CO adsorption on anatase TiO_2_ surfaces, focusing on the (001) and (111) facets, employing CO as an IR probe. Our results reveal a substantial reconstruction of the (111) surface from its idealized structure. Notably, we predict that this surface will give rise to two distinct IR signals corresponding to two chemically different surface sites. The analysis shows that adsorption behavior and associated vibrational shifts are strongly influenced by surface geometry and the local coordination environment of Ti atoms.

On the (111) facet, CO interacts differently depending on the site: σ-donation is dominant at the [TiO_3_] site, whereas π-backdonation prevails at the [TiO_5_] site, modulated by the presence of nearby oxygen atoms. Vibrational frequency predictions indicate an 8 cm⁻^1^ blue shift for CO adsorbed on the (001) surface and both a 43 cm⁻^1^ blue shift and a 7 cm⁻^1^ red shift for CO on the (111) surface, depending on the adsorption site. Overall, our findings highlight the importance of surface topology—not just Ti coordination—in determining the adsorption characteristics of probe molecules like CO.

## Supplementary Information

Below is the link to the electronic supplementary material.

ESM 1 (DOCX 1.34 MB )

## Data Availability

Vacek, Jaroslav (2026), “Theoretical Study of TiO2 Anatase Surfaces via a CO IR Probe.”, Mendeley Data, V3, 10.17632/fvf5khr284.3 (https://data.mendeley.com/datasets/fvf5khr284/3).
