# Supplementary material for: Mechanistic insights into facet-dependent CO adsorption and vibrational responses on anatase TiO2: periodic quantum-mechanical calculations
Source: J Mol Model. 2026 Apr 13;32(5):128. doi: 10.1007/s00894-026-06703-w (PMC13076413; doi:10.1007/s00894-026-06703-w)
Supplement: Supplementary file 1 — (DOCX 1.34 MB ) [file 894_2026_6703_MOESM1_ESM.docx]

**Suporting Information**

**Mechanistic Insights into Facet-dependent CO Adsorption and Vibrational Responses on Anatase TiO₂: Periodic Quantum-Mechanical Calculations.**

Jaroslav Vacek^1,2,3,^[^iD^](https://orcid.org/0009-0008-7075-9668) , Pavel Hobza^1,2,^[^iD^](https://orcid.org/0000-0002-9588-8625), Dana Nachtigallová^*1,2,^ [^iD^](https://orcid.org/0000-0001-5292-6719)

^1^Institute of Organic Chemistry and Biochemistry, Czech Academy of Sciences, Flemingovo námĕstí 542/2, 16000 Prague, Czech Republic

^2^IT4Innovations, VŠB − Technical University of Ostrava, 17. listopadu 2172/15, 708 00 Ostrava-Poruba, Czech Republic

^3^Department of Physical Chemistry, Palacký University Olomouc, tř. 17. listopadu 12, 771 46 Olomouc, Czech Republic

**Correspondence:** Dana Nachtigallová ([dana.nachtigallova@uochb.cas.cz](mailto:dana.nachtigallova@uochb.cas.cz))

*Model details*

A 2x2 super cell vase used for the 001 surface, the slab was about 12 A thick (12.59 A) with a layer of 20 A of vacuum. The cell has a volume of 1853.55 Å³, the vectors were 7.57 Å, 7.57 Å, 32.36 Å (a, b and c respectively), with angles 90.00 °, 90.00 °, and 90.00 (alpha, beta and gamma respectively). The cell contains 24 TiO_2_ units (24 Ti atoms and 48 O atoms).

The (111) surface was modelled by a 2x2 orthorhombic cell. As seen in Table S1, it is shown that this supercell is sufficient, while being practically small. The slab is about 12 A thick (11.85 A). As mentioned in the main text, the surface undergoes a large reconstruction when optimised. This leads to a slight widening of the slab, resulting in a slab of a thickness of 12.51 A. The cell has a volume of 6748.31 Å³, the vectors were 10.70 Å, 19.77 Å, 31.90 Å (a, b and c respectively), with angles 90.00 °, 90.00 °, and 90.00 (alpha, beta and gamma respectively). The cell contains 72 TiO_2_ units (72 Ti atoms and 144 O atoms).

| Table S1: Comparison of Reconstruction energies of different supercells of the surface of (111) of anatase. All Slabs have the same thickness; they differ in volume and shape. The vector c is thus constant; vectors a and b are different numbers of primitive vectors. The 2x2 orthorhombic cell is equivalent in volume to the 2x2 cell. | | |
| --- | --- | --- |
| Supercell of the (111) surface | TiO_2_ Units | Energy of Reconstruction per TiO_2_ Unit / kcal mol^-1^ |
| 1x1 | 18 | -17.99 |
| 2x2 | 72 | -18.53 |
| 2x2 orthorhombic | 72 | -18.52 |
| 2x3 | 108 | -10.93 |
| 3x2 | 108 | -18.46 |
| 3x3 | 162 | -18.46 |
| 4x4 | 288 | -18.46 |

*Benchmark calculations*
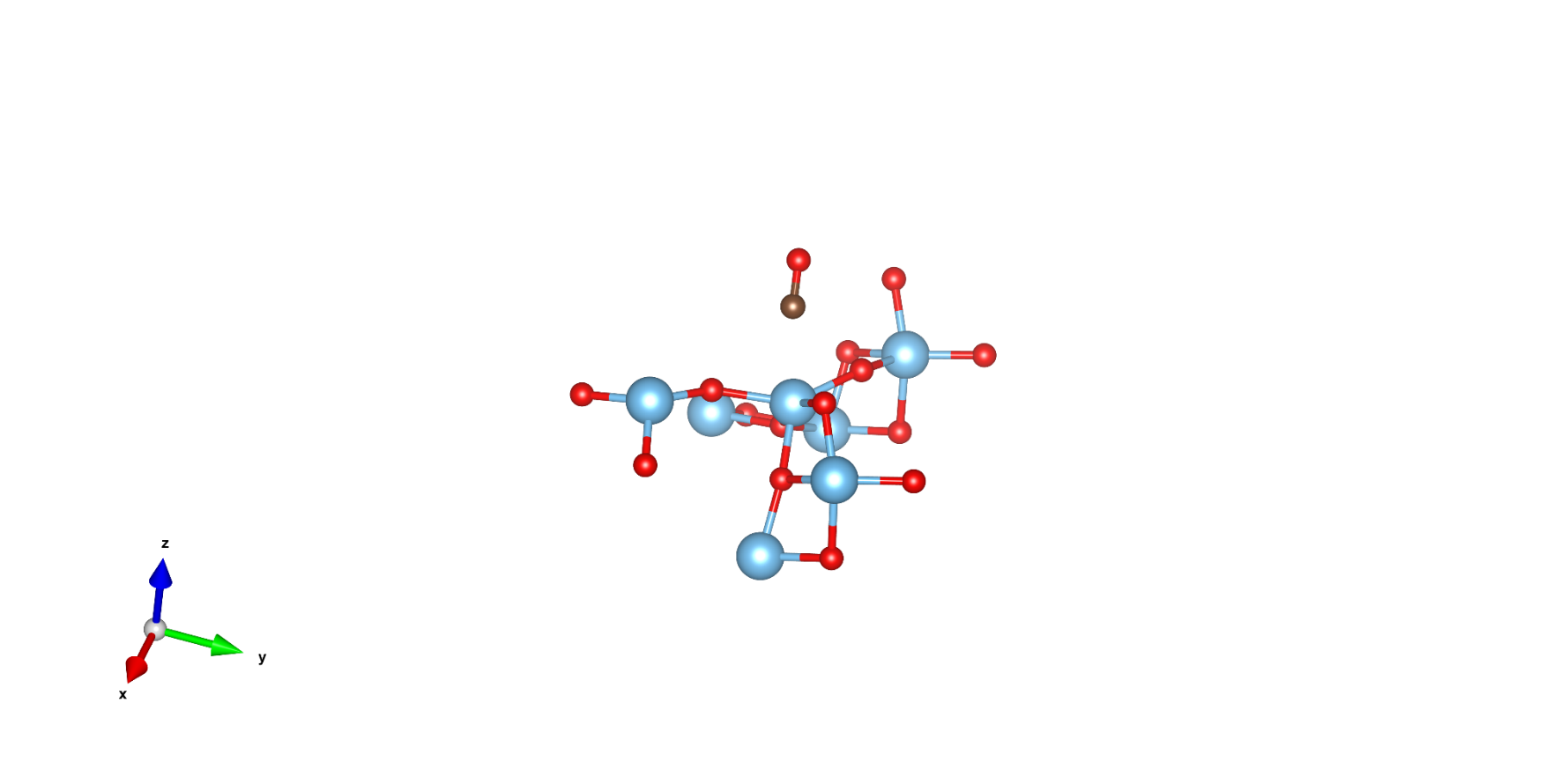

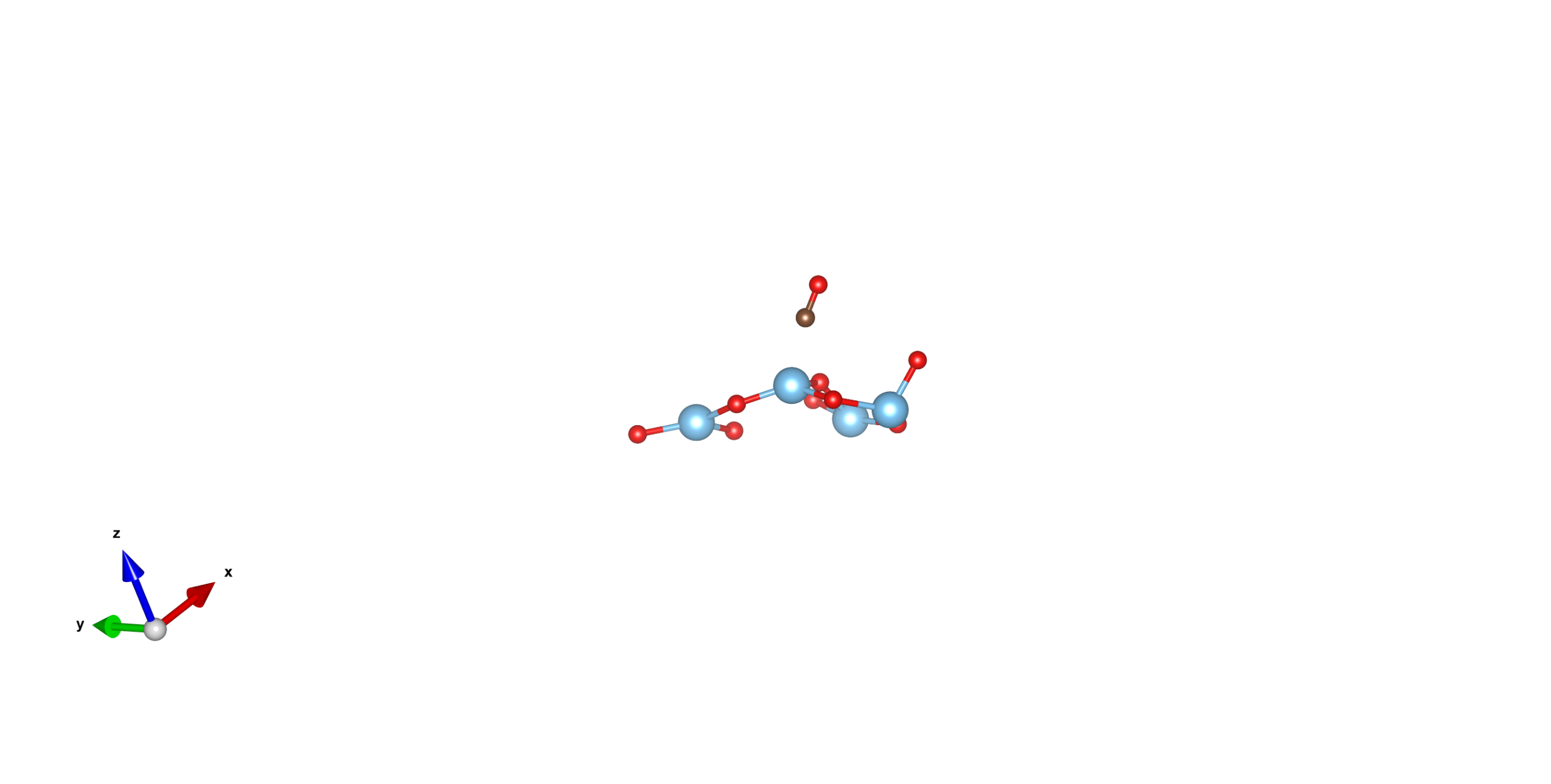


A

B

Figure S1: The minimal models of the 111 surface used for benchmarking. The models are centred around a triple-coordinated Ti atom (A) and a pentacoordinate Ti atom (B). The models are stoichiometric. The CO molecules are adsorbed on the respective sites.

| Table S2: The CO bond distances (r_CO_, Å) and their changes upon bonding (Δr_CO_, Å), and CO vibrational frequencies (ν_CO_, cm^-1^) and their changes upon bonding (Δν_CO_ , cm^-1^) calculated on CO-Ti-(O-Ti)_n_-O_X_^a^ models of (111) anatase using DFT and MP2 methods and TZVP basis set. | | | | |
| --- | --- | --- | --- | --- |
|  |  | CO | 111 [TiO_3_] | 111 [TiO_5_] |
| r_CO_/Δr_CO_ | PBE | 1.1382 | 1.1336/-0.0046 | 1.1369/-0.0013 |
|  | B3LYP | 1.1269 | 1.1179/-0.0090 | 1.1239/-0.0030 |
|  | MP2 | 1.1389 | 1.1318/-0.0071 | 1.1369/-0.0020 |
| ν_CO_/Δν_CO_ | PBE | 2133 | 2145/12 | 2136/3 |
|  | B3LYP | 2217 | 2294/77 | 2238/21 |
|  | MP2 | 2129 | 2191/61 | 2160/31 |
| ^a^n = 3 or 5, X denotes the number of oxygen atoms maintaining the TiO_2_ stoichiometry | | | | |

Table S2 presents a comparison of partial cluster optimization results for CO-Ti-(O-Ti)_n_-O_X_ clusters (n = 3 or 5, X denotes the number of oxygen atoms to keep the TiO_2_ stoichiometry) clusters of anatase 111 (Figure S1) using the DFT and MP2 methods. In these calculations, only the CO bond distance (r), and stretching vibrational frequency (ν) were optimized, while the surface atoms remained fixed at the geometry derived from the crystal-optimized structure.


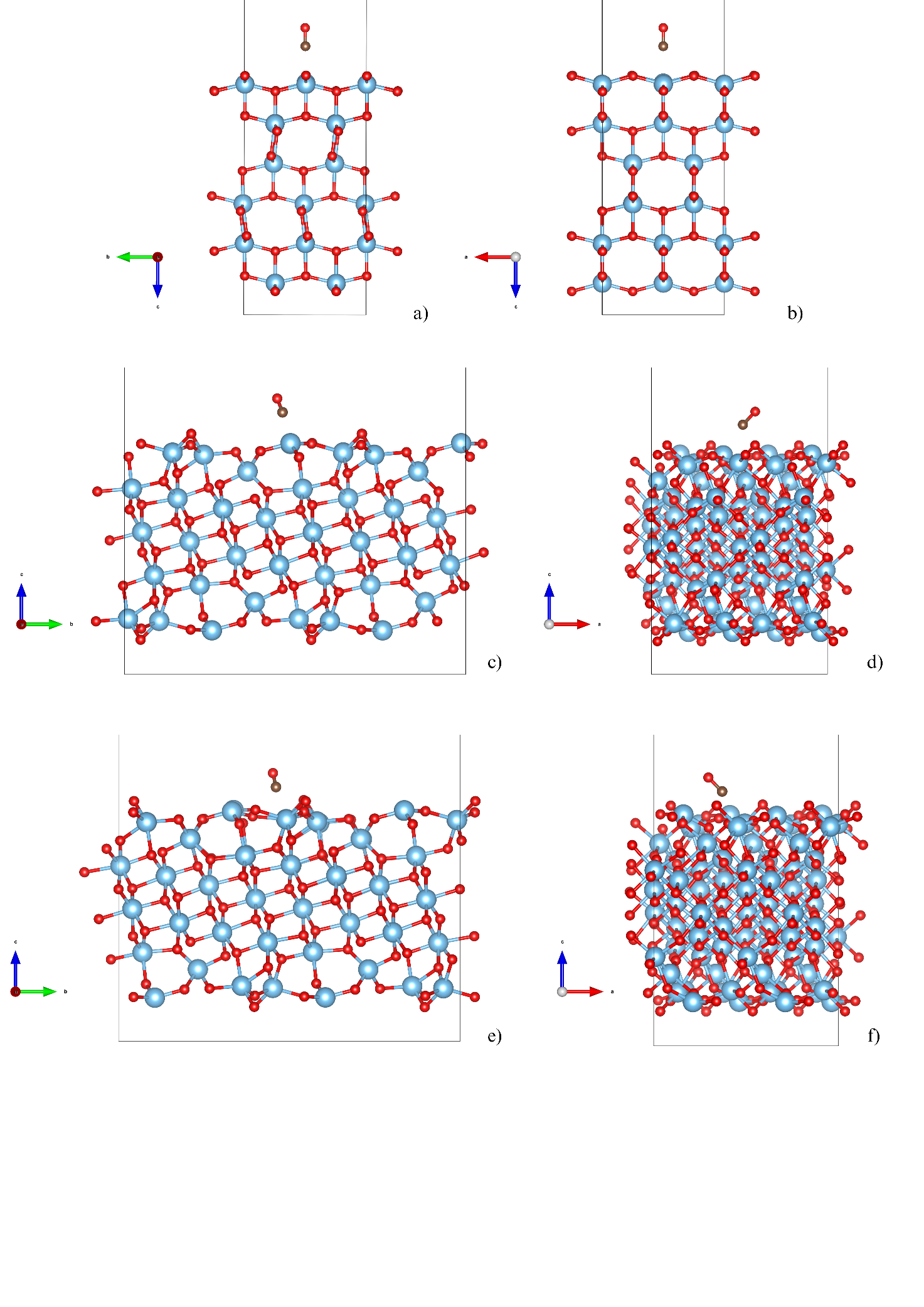


Figure S2: The CO molecule adsorbed on the three sites as seen from two crystallographic directions, a) CO-001 [TiO_5_] in a axis direction, b) CO-001 [TiO_5_] in b axis direction, c) CO-111 [TiO_3_] in a axis direction, d) CO-111 [TiO_3_] in b axis direction, e) CO-111 [TiO_5_] in a axis direction and CO-111 [TiO_5_] in b axis direction.

*Computational details*

1.1 The periodic boundary calculation.

All periodic boundary condition calculations were performed by the Viena Ab-initio Simulation Paced (VASP)[1, 2]. The kinetic energy cut-off was set at 520 eV. As all supercells were quite large, the Gama point sampling was sufficient. Only for the DOS and total electron density calculation was the number of K-points interest to a 7-7-1 gride. The core electrons were described by the Projector-augmented-wave formalism[3].

The analysis of the DOS data was done via the VASPKIT script[4]. VAPKIT was also used to decompose the electronic band structures. The two-electron band structures and the difference of electron densities were visualized by the VESTA program.

The difference of the electronic densities $\Delta\rho\left( CO-Surface \right)$ is defined in Eq. S1.:

$\Delta\rho\left( CO-Surface \right)= \rho\left( CO-Surface \right)-[\rho\left( Surface \right) + \rho\left( CO \right)]$ Eq. S1

Where $\rho\left( CO-Surface \right)$ is the total electronic density of the CO-Surface complex, $\rho\left( CO \right)$is the total electronic density of an isolated CO molecule and $\rho\left( Surface \right)$ is the total electronic density of the isolated surface.

1.2 Model preparation.

The slab models were prepared, from the experimental data, via the build tool of QantumATK software [5].

All non-periodic cluster calculations were done via the ORCA package[6].

1. Kresse G, Hafner J (1993). Ab initio molecular dynamics for liquid metals. Physical Review B 47:558

2. Kresse G, Furthmüller J (1996). Efficient iterative schemes for ab initio total-energy calculations using a plane-wave basis set. Phys Rev B 54:1116

3. Joubert D (1999). From ultrasoft pseudopotentials to the projector augmented-wave method. Phys Rev B 59:1758

4. Wang V, Xu N, Liu JC, Tang G, Geng WT (2021). VASPKIT: A user-friendly interface facilitating high-throughput computing and analysis using VASP code. Comput Phys Commun 267:108033

5. Smidstrup S, Markussen T, Vancraeyveld P, Wellendorff J, Schneide J, Gunst T, Stokbro K (2020). QuantumATK: An integrated platform of electronic and atomic-scale modelling tools. J Phys Condens Matter 32:015901

6. Neese F (2012). The ORCA program system. Wiley Interdiscip Rev Comput Mol Sci 2:73–78
